# Hyperemesis gravidarum in northern Israel: a retrospective epidemiological study

**DOI:** 10.1186/s13584-016-0100-9

**Published:** 2016-10-01

**Authors:** Tom Konikoff, Tehila Avraham, Ella Ophir, Jacob Bornstein

**Affiliations:** 1 Faculty of Medicine in the Galilee, Bar Ilan University, Safed, Israel; 2 Department of Statistical Studies, Hebrew University, Jerusalem, Israel; 3 Department of Obstetrics and Gynecology, Galilee Medical Center, Nahariya, Israel; 4 Department of Internal Medicine D, Rabin Medical Center Beilinson Hospital, Petach Tikva, 4941492 Israel

**Keywords:** Hyperemesis gravidarum, Arab-Jewish population comparison, Pregnancy complications, Economic burden of disease

## Abstract

**Background:**

Hyperemesis gravidarum (HG) is characterized by severe intractable nausea and vomiting in pregnancy leading to electrolyte imbalance, ketonuria, and weight loss. The cause is unknown. This study sought to investigate the prevalence and characteristics of HG in the Western Galilee in two ethnic populations and to estimate its economic burden.

**Methods:**

Data on ethnicity, age, gestational age, number of pregnancies, and length of hospitalization were collected from the medical files of all women with HG admitted to the Galilee Medical Center in 2010–2013. Findings were compared between Arabs and Jews. Prevalence was assessed relative to total number of births. Economic burden was assessed by cost of hospitalization and work days lost.

**Results:**

The cohort included 184 women, 124 Arabic (67.4 %) and 60 Jewish (32.6 %). There were 13,630 births at the medical center during the study period, for a calculated prevalence of HG of 1.2 %. There was no difference in the relative proportions of Arabs and Jews between the cohort and the total women giving birth at our center. Mean patient age was 27.2 years, gestational age 9.3 weeks, parity 2.35. Mean age was significantly higher in the Jewish group. There were no significant between-group differences in the other clinical parameters. Mean number of hospitalization days was 2.24 days, and of additional rest days prescribed, 4.62. The calculated annual cost of HG was 452,943.42 NIS (120,144.14 USD), crudely extrapolated to a nationwide cost of 15–20 million NIS (5,300,000 USD).

**Conclusion:**

The prevalence and characteristics of HG are similar in the Arabic and Jewish populations of northern Israel. Mean gestational age at admission for HG was lower in our study than earlier ones, probably owing to the universal health care provided by law in Israel. HG prevalence was twice that reported previously in southern Israel but still within the range observed in other world regions. The socioeconomic differences between Arabs and Jews in the Galilee are smaller than elsewhere in Israel, suggesting a multifactorial etiology of HG. HG poses a major economic burden which should be considered when planning health policies. Further studies of this issue are warranted.

## Background

Approximately 50 % – 90 % of women experience nausea and vomiting during pregnancy [1]. A much smaller percentage, ranging from 0.3 % to 2.0 %, are diagnosed with hyperemesis gravidarum (HG), a type of severe intractable vomiting accompanied by dehydration, ketonuria, electrolyte disturbance, and weight loss of more than 5 % of the prepregnancy weight [1]. HG typically appears between 3 and 5 gestational weeks and resolves by 20 weeks [2]. Besides its high morbidity, HG poses a considerable economic burden in terms of hospitalization days and loss of work days for otherwise healthy functional persons [3]. The cause is unknown, although several studies have putatively implicated high human chorionic gonadotropin levels [4], hyperthyroidism [5], and *Helicobacter pylori* infection [6]. HG has also been associated with female fetuses [7]. However, despite the possible metabolic component of HG, very few studies have compared prevalence rates among ethnic groups, given their known differences in food consumption and lifestyle, or investigated the possible role of environmental factors. Most of the ethnicity-related findings in HG were secondary to investigations of fetal outcome and included a significantly high incidence in non-white populations (33 % vs 16 % in white populations; *p* <0.05) [8] and east-Asian populations (3.6 %) [9]; others noted no difference between specific ethnic groups [10, 11]. The only study of HG that focused specifically on ethnicity, conducted in Norway, showed that immigrants were at higher risk than native Norwegians [12], possibly pointing to the involvement of genetic factors as well.

The widely diverse population of northern Israel, especially the Galilee, includes several ethnic groups with unique genetic makeup. The Jews and Arabs who inhabit the region have many distinct cultural and lifestyle habits. For instance, Arabs consume high levels of olive oil in addition to meat, particularly lamb, and other fatty foods compared to Jews. A higher proportion of Arab men than Jewish men are smokers, and consequently, a higher proportion of Arab women than Jewish women are exposed to second-hand smoke [13]. Although the exact rates of these parameters in Arabs and Jews and their various subpopulations (e.g., Ashkenazi/Sephardic Jews; Muslim/Christian Arabs, Druze), are difficult to determine, they may at least partly explain the relatively high prevalence of the metabolic syndrome in Israeli Arabs [13].

To date, the only study of the incidence of HG in Israel, overall and by ethnicity, was conducted in Soroka Medical Center situated in the southern city of Beer Sheva [14]. Findings were compared between the Jewish and Bedouin populations, with the Jewish population showing a significantly higher incidence of HG. However, the Arab populations of southern and northern Israel differ greatly in terms of genetics, culture, and socioeconomic factors. For example, the residents of the city of Rahat in the south are exclusively Muslim Bedouin. The rate of individuals with an academic education is very low, and the average socioeconomic grade is 1/ 10 (Rahat) [15].

By contrast, the residents of the Arab town of Mi’ilya in the Galilee are almost exclusively Catholic. More than 30 % have an academic education and the average socioeconomic grade is 6/10 [15].

The present study was conducted in the Department of Obstetrics and Gynecology of the Galilee Medical Center in Nahariya, the largest such facility in northern Israel, serving a catchment area of 570,000 residents. More than 5000 births take place here each year, the highest number north of Tel Aviv. The aim of the study was to investigate the prevalence and characteristics of HG among women living in the northern region of the Galilee, with comparison between Jews and Arabs. We also examined its economic burden in terms of hospitalization costs and, as HG affects women of reproductive age who are usually working, in lost work days. We hypothesize that there is a significant difference in the prevalence of HG between these two ethnic populations, which would suggest a multifactorial etiology, including lifestyle habits and a possible genetic component. Second, we hypothesize that HG constitutes a large burden on the Israeli economy.

## Methods

A retrospective population-based epidemiological study design was used. The database of the Department of Obstetrics and Gynecology of Galilee Medical Center, Nahariya, Israel was reviewed for all women admitted with a diagnosis of HG from December 1, 2010–December 31, 2013. Data on ethnicity (Jewish/Arabic), age, gestational age (in weeks) at admission, number of pregnancies and number of hospitalization days were collected from the medical files. Each woman admitted counted as a single case, regardless of the number of her hospitalizations for HG during the study period. Criteria for the diagnosis of HG were intractable vomiting, dehydration, electrolyte disturbance, and ketonuria. According to hospital policy, women who present with HG are referred to the Department of Obstetrics and Gynecology, so the cases of HG included in the study represent 100 % of all cases seen in the medical center during the study period. The study protocol was approved by the local Institutional Review Board (Helsinki Committee).

The data were summarized using Microsoft Excel and processed statistically using a binominal test. To determine the prevalence of HG, the number of cases of HG was calculated as a proportion of the total number of births at our center during the same period, overall and by ethnic group. Independent *t*-test was used to compare age at admission between Jewish and Arab women. Nonparametric Mann-Whitney test was used to compare the groups for gestational age at admission and number of hospitalization days. A p value of <0.05 was considered statistically significant. The economic burden of HG (P), we calculated the average length of hospitalization (d), average number of additional rest days prescribed at discharge (r), price of a single hospitalization day (c), average employee daily wage (w) And rate of unemployment (e). These parameters were used to build the following equation: p = e*(d + r)*w + (d*c).

According to Israeli law, the state is responsible for providing health services, including hospitalization, to all residents of the country, who may register with any one of the four health maintenance organizations (HMOs). The cost of hospitalization in public hospitals is determined by the Israel Ministry of Health and paid by the HMOs, who submit an annual report to the ministry. The average wage in the Galilee for women and the unemployment rate during the study period were derived from the most recent (2011) published data of the Israel Central Bureau of Statistics.

## Results

The cohort included 184 women with HG, 124 Arabic (67.4 %) and 60 Jewish (32.6 %). There were 13,630 births at the medical center during the study period, for a calculated prevalence of HG of 1.2 %. There was no significant difference in the relative proportions of Arabic and Jewish women between the study group and the total population of women who gave birth during the study period (Arabic: *n* = 8586, 63 %; Jewish: *n* = 5043, 37 %; 0.189).

The demographic data of the patients are summarized in Table 1. Mean (±2SD) values for the cohort were age 27.2 years (range 16–44), gestational age 9.2 weeks (range 5–17.5), and number of pregnancies 2.35 (range 1–4). On analysis by ethnicity, the Jewish group was significantly older than the Arabic group (28.9 ± 10.6 years vs 26.4 ± 10.2 years, *p* = 0.002). There were no significant differences between the Arabic and Jewish groups in mean (±2SD) gestational age (9.3 ± 4.8 weeks vs 9.2 ± 5.8 weeks, *p* = 0.478) or number of pregnancies (2.2 ± 2.8 vs 2.5 ± 4.2, *p* = 0.298). The mean (±2SD) number of hospitalization days was 2.2 ± 2.2 in the Arabic group and 2.2 ± 3.4 in the Jewish group with a smaller range of hospitalization days for Arab women (*p* = 0.034).

**Table 1:** Background parameters of 184 women hospitalized for HG, total and by ethnicity

Parameter	Total cohort	Arabs	Jews	*P* value (Arabs vs Jews)
No. patients (%)	184	124 (67.4 %)	60 (32.6 %)	
Age (yr), mean ± 2SD (range)	27.3 ± 10.6 (16-44)	26.4 ± 10.2 (16-40)	28.9 ± 10.6 (16-44)	0.002 (*t*-test)
Gestational age (wk), mean ± 2SD (range)	9.28 ± 5.2 (5-17.5)	9.3 ± 4.8 (5-17)	9.2 ± 5.8 (5.5-17.5)	0.478 (Mann-Whitney)
No. of pregnancies, mean ± 2SD (range)	2.35 ± 3.4 (1-4)	2.2 ± 2.8 (1-4)	2.5 ± 4.2 (1-4)	0.298 (chi-square)
Length of hospitalization (days), mean ± 2SD (range)	2.24 ± 2.6 (1-9)	2.2 ± 2.2 (1-6)	2.2 ± 3.4 (1-9)	0.034 (Mann-Whitney)

Analysis of the economic burden showed that HG accounted for an annual average of 137.6 hospitalization days. The average number of rest days prescribed per patient was 4.62, for an annual average of 283.36. The price of one day’s hospitalization day in the Department of Obstetrics and Gynecology during the study period was 2999 NIS [16]. According to the most recent (2011) estimate, the average daily wage for women in the region was 292.74 NIS [17], and the estimated employment rate for women in the Galilee was 55.2 % [18]. Therefore, using our equation, the estimated cost of HG in the Western Galilee of Israel during the study period was 452,943.42 NIS (120,144.14 USD, 3.77 NIS/1 USD). Hospitalization accounted for the bulk of this amount (412,662.4 NIS), paid by the health service funds, and lost work days for the remainder (40,281.02 NIS), paid by employers (given that employers often arrange for substitutes for absentee employees at the same daily wage). Considering that the total annual number of births in Israel is much higher than in the Western Galilee alone, reaching 170,940 in 2013 [19], the estimated national annual cost of HG may reach more than 15 million NIS. Moreover, the unemployment rate in the Western Galilee is significantly higher than central Israel [20], and the average daily wage is significantly lower [20]. If we adjust the figures to central Israel, taking higher employer losses and additional expenses (such as spouses taking work leave to attend to children at home) into account, the figure may easily rise to 15–20 million NIS per year (5,300,000 USD).

## Discussion

The present study describes the characteristics of HG in the Western Galilee region of Israel, its possible association with ethnicity (Arabic/Jewish), and its economic impact. The possible connection of HG to ethnicity has hardly been investigated. The major studies conducted to date are shown in Table 2. Our search of the medical literature yielded no studies on the role of environmental factors in HG.

**Table 2:** Summary of studies evaluating the connection between HG and ethnicity

Study	Study population	Ethnicity in aim of study	Ethnic groups assessed	HG prevalence
Vilming and Neshem [10], Norway (1993-1997)	*N* = 120	Primary	Norwegian, non-Norwegian	Higher in non-Norwegian
Paauw et al [20], USA (1984-1991)	*N* = 45	Secondary	Whites, non-White	Higher incidence in non-Whites
Vlachodimitropoulou-Koumoutsea et al [9], UK (2007-2010)	*N* = 208	Secondary	Caucasian, African/Caribbean, Asian, Oriental, mixed	No significant difference
Bailit [2], USA (1999)	*N* = 2,466	Secondary	White, Non-White, Hispanic	Higher in Non-Whites
Bashiri et al [4], Israel (19985-1988)	*N* = 164	Secondary	Jewish, Bedouin	Higher in Jewish
Konikoff et al. Israel (2010-2013)	*N* = 184	Primary	Jewish, Arab	No significant difference

The main finding of the present study is that HG has a prevalence rate of 1.2 % in the Western Galilee. This is twice the rate reported by Bashiri et al [14] in southern Israel (0.6 %) but still within the accepted range of the proportion of pregnancies complicated by HG (0.3–2 %) [2] and similar to other regions in the world [1, 2].

Our analysis showed that Jewish women with HG are significantly older than Arabic women with HG. This finding was not unexpected given the general cultural differences between the populations [21]. Whether patient age affects the incidence of HG is unclear. Others have shown that HG is more prevalent in younger women [1].

The average gestational age at admission for HG (9.2 weeks). is considerably lower than the average reported by a large epidemiologic study on HG from California (11.3 gestational weeks) [2]. The difference may be explained by the mandatory medical coverage of all residents in Israel as opposed to the largely private healthcare system in the United States, which makes patient referral for hospitalization considerably easier in Israel.

The average length of hospitalization for HG in our study (2.24 days) is similar to findings in the study from California (2.6 days) [2].

The relative proportions of Jewish and Arabic women hospitalized for HG in our cohort reflect the ethnic composition of the population of the Western Galilee. The lack of a significant difference between the Arab and Jewish groups in the proportion of HG cases out of total births does not agree with our hypothesis. We find it surprising given the distinct genetics and lifestyles of the two groups as well as the higher incidence of HG in Jewish than in Bedouin women reported by Bashiri et al [14] in southern Israel. These authors suggested that the poorer and less educated Bedouin women may not have presented for treatment because of a lower awareness of the serious complications of HG than the Jewish women. Alternatively, they may have a true low prevalence for unknown reasons. In the Western Galilee, the Arab population has a lower socioeconomic status than the Jewish population, but it is still higher than that of the southern Bedouin. Accordingly, in our study, there was no between-group difference in parity. Furthermore, although the Arab population of the Western Galilee is mainly Muslim and has a high rate of consanguineous marriage whereas the Jewish population is made up mainly of individuals of European descent or new immigrants from the former Soviet Union, the proportion of Jews of Middle Eastern and north African descent is steadily growing. This may have increased the genetic and lifestyle similarity of the Jewish and Arab groups. Owing to the retrospective nature of this study, we were unable to determine the exact proportion of Jews of European, Middle Eastern, and North African descent. These factors require additional in-depth investigations.

The present study highlights the economic burden of HG in Israel, reaching almost half a million shekels annually in the Western Galilee alone. Most of the cost is carried by the HMOs. The crudely estimated cost for the entire country, taking into account the denser population, higher employment rate, and higher average salaries in central Israel, is15–20 million shekels (5,300,000 USD). Thus, HG must be considered in health services planning.

This study is limited by its retrospective design. Additionally, the assessment of national trends based on regional studies in Israel is difficult owing to the wide diversity of the populations in terms of cultural background, lifestyle habits, socioeconomic status, educational level, and quality of medical care. Studies are needed comparing HG between the central and peripheral areas of the country and among the various subpopulations.

## Conclusions

The overall prevalence of HG in the Western Galilee is 1.2 %, similar to reports in other regions and countries. The similar prevalence rates of HG in the region’s Arabic and Jewish populations, which have widely diverse national, cultural, and lifestyle backgrounds, may suggest a multifactorial etiology. HG exerts a considerable economic burden, mainly on the healthcare system and also on the financial system, as it affects young women who make up a considerable proportion of the working public. This issue, which has never been addressed, has major implications for increasing the number and quality of ambulatory community clinics in the area, especially in the poorer sectors, and raising awareness of the syndrome and the importance of its early management among both the public and medical professionals. Many question remain unresolved, and further investigations are warranted.

## Abbreviations

HG: Hyperemesis gravidarum
HMO: Health maintenance organization
NIS: New Israeli Sheqel
USD: United states dollar
